# Immune Checkpoint Inhibitor-Induced Hepatitis: A Case Report of Challenging Management

**DOI:** 10.7759/cureus.77331

**Published:** 2025-01-12

**Authors:** Inês Soldin, Raquel Teixeira, Raquel Ortigão, Alexandra Lapa, Sérgio Lima

**Affiliations:** 1 Medical Oncology, Portuguese Institute of Oncology Francisco Gentil, Porto, PRT; 2 Gastroenterology, Portuguese Institute of Oncology Francisco Gentil, Porto, PRT; 3 Pathology, Portuguese Institute of Oncology Francisco Gentil, Porto, PRT; 4 Internal Medicine, Portuguese Institute of Oncology Francisco Gentil, Porto, PRT

**Keywords:** checkpoint inhibitor-induced liver injury, immune-checkpoint inhibitors, immune-related adverse events, lung cancer, steroids and immunosuppressants

## Abstract

Pembrolizumab is an immune checkpoint inhibitor (ICI) that is demonstrated to enhance the prognosis of patients with advanced lung cancer. However, adding immunotherapy to clinical practice has brought new challenges, such as immune-related adverse events (irAEs), which have changed chemotherapy's previously well-understood safety profile. Immune-mediated hepatitis, although less prevalent and less extensively studied, represents a significant toxicity that may evolve into a potentially severe complication, particularly when it becomes refractory to conventional treatments. In this report, we present the case of a 67-year-old male patient with non-small cell lung cancer who developed severe corticosteroid (CS)-refractory hepatitis following two cycles of pembrolizumab. Differential diagnosis workup excluded alternative diagnosis. A liver biopsy evidenced both hepatitis and cholestasis. Due to persistent cytolysis, it was necessary to add mycophenolate mofetil (MMF). Additionally, ursodeoxycholic acid (UDCA) was introduced due to persistent cholestasis, resulting in the normalization of laboratory parameters. The lack of prospective evidence regarding immune-related hepatitis treatment makes it challenging to draw definitive conclusions about the optimal therapeutic approach.

## Introduction

Pembrolizumab, an immune checkpoint inhibitor (ICI), is a monoclonal antibody that targets the programmed cell death-1 (PD-1) inhibitory receptor on the surface of T cells [1]. ICIs may induce multisystem immune-related adverse events (irAEs), affecting up to 90% of treated patients, depending on the specific immune checkpoint targeted and drug combination [1]. Among these irAEs, checkpoint inhibitor-induced liver injury (ChILI) is observed in 5-10% of patients receiving ICI monotherapy, typically presenting as asymptomatic elevation of liver enzymes. Severe hepatitis (Common Terminology Criteria for Adverse Events (CTCAE) grade ≥3) implies ICI discontinuation and occurs in less than 5% of cases [1,2].

The median time to hepatitis onset following PD-L1 inhibitor administration is approximately 8-12 weeks, with delayed-onset hepatitis being uncommon [1,2]. ChILI exhibits considerable heterogeneity, as has been increasingly documented [1]. Based on the ratio R (ALT/ULN)/(ALP/ULN), ChILI can manifest in a cholestatic pattern (frequently associated with monotherapy using anti-PD-L1 inhibitors), hepatocellular (more prevalent in combination therapies involving anti-cytotoxic T-lymphocyte-associated antigen 4 inhibitors and correlating with hepatitis severity), and mixed presentations. The cholestatic subtype typically demonstrates a moderate to poor response to corticosteroids (CS) but may benefit from treatment with ursodeoxycholic acid (UDCA) [1,3-5].

For cases of severe hepatitis, guidelines recommend permanent discontinuation of ICIs and initiation of CS (1-2 mg/kg/day methylprednisolone) [2,5]. Multidisciplinary consultation, including a hepatologist, is essential. European Association for the Study of the Liver recommends performing a liver biopsy in severe ChILI cases or refractory hepatotoxicity [2,6]. In CS-resistant hepatitis, alternative immunosuppressive therapy should be initiated within two to three days, with options including mycophenolate mofetil (MMF) [2,6,7].

The clinical heterogeneity of ChILI challenges the formulation of updated guidelines for ICI rechallenge [1]. However, given that ICI-treated patients often present with advanced-stage cancer with limited treatment options, the decision to discontinue immunotherapy permanently is a reasonable clinical dilemma [7].

## Case presentation

We present the case of a 67-year-old male diagnosed with pulmonary non-small cell carcinoma with pleomorphic features in the right lower lobe, staged as cT3N2M1 (stage IV), and strong PD-L1 expression (90-100%). A KRAS G12C mutation was identified through genetic analysis. Clinically, the patient had an Eastern Cooperative Oncology Group (ECOG) Performance Status of 1 at diagnosis.

Concerning medical antecedents, he has a history of heart failure with reduced ejection fraction, secondary to valvular (severe mitral insufficiency) and arrhythmogenic causes (atrial fibrillation), managed with standard guideline-directed medical therapy. He was also on warfarin, with the International Normalized Ratio (INR) normally on target.

He is a former heavy smoker (100 pack-years) and had a history of significant alcohol consumption (124 g of alcohol/day), which he had reduced to 20 g of alcohol/day at the time of oncological diagnosis. In this context, the patient also had a prior diagnosis of chronic liver disease of alcoholic etiology, without stigmas of cirrhosis. No additional past medical history or concurrent medication was notable.

Given the stage IV malignancy with high PD-L1 expression, favorable ECOG status, and absence of contraindications, pembrolizumab 200 mg every three weeks was initiated in August 2024.

On day two of the second treatment cycle, the patient presented a markedly elevated INR of 11.63 on a routine INR assessment. The patient denied wrong warfarin intake, recent medication changes, use of over-the-counter drugs or supplements, and alcohol abuse. He reported no symptoms suggestive of bleeding. Physical examination revealed mild jaundice and bilateral lower limb edema, with no signs of active bleeding or other forms of coagulopathy. Laboratory evaluation showed grade 4 aspartate aminotransferase (AST) and alanine aminotransferase (ALT) increased (4314 U/L and 3557 U/L, respectively), hyperbilirubinemia (total bilirubin: 4.22 mg/dL; direct bilirubin: 2.64 mg/dL), grade 3 gamma-glutamyl transferase (GGT) elevation (324 U/L), and grade 1 alkaline phosphatase (ALP) elevation (312 U/L) with a normal albumin level (3.8 g/dL). Coagulation studies confirmed an elevated INR of 14.68 (Table 1). Both complete blood count and arterial blood gas were normal. A diagnosis of acute grade 4 hepatitis was assumed.

**Table 1:** Liver function workup ALT, alanine aminotransferase; AST, aspartate aminotransferase; GGT, gamma-glutamyl transferase; ALP, alkaline phosphatase; INR, International Normalized Ratio

Parameter	Minimum	Maximum	Reference range (unit)
ALT	124	3557	<42 U/L
AST	47	4314	<39 U/L
GGT	308	782	7-52 U/L
ALP	133	312	42-128 U/L
Total bilirubin	4.22	17.85	<1 mg/dL
Direct bilirubin	2.64	13.50	<0.25 mg/dL
Albumin	2.8	3.8	3.8-5.3 g/dL
INR	1.14	14.68	0.9-1.2

The patient was admitted and empirical methylprednisolone at 2 mg/kg/day was initiated due to suspected immune-mediated hepatitis, given the severity of the presentation.

In anticipation of prolonged immunosuppression, latent tuberculosis was ruled out, and prophylactic cotrimoxazole 960 mg three times a week was initiated.

Laboratory evaluation for differential diagnosis revealed negative results for hepatitis A, B, and C and excluded de novo infection or reactivation of other hepatotropic viruses, including Epstein-Barr, parvovirus, and cytomegalovirus. Testing for human immunodeficiency virus and syphilis was also negative. An autoantibody panel (including antibodies to nuclear, smooth muscle, and liver-kidney microsomal antigens) was negative, excluding autoimmune hepatitis. Hormonal studies including thyroid and adrenocortical function were within normal limits.

**Figure 1:**
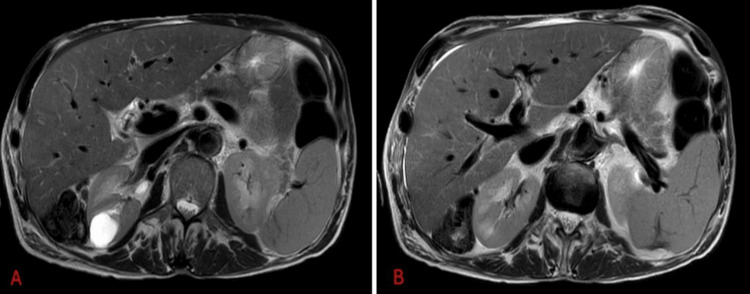
MRI of the liver A: T1 weighted; B, T2: weighted; A/B: Liver with slight heterogeneity of parenchymal texture, related to suspicion of hepatitis. Multiple liver hilar lymphadenopathy, with short axes that do not exceed 7 mm. Splenomegaly, measuring the spleen 174 mm in the greatest axis. No other relevant changes. MRI, magnetic resonance imaging

Abdominal ultrasound and magnetic resonance imaging (MRI) ruled out liver disease progression (Figure 1). A Doppler ultrasound study excluded portal and supra-hepatic vein thrombosis.

**Figure 2:**
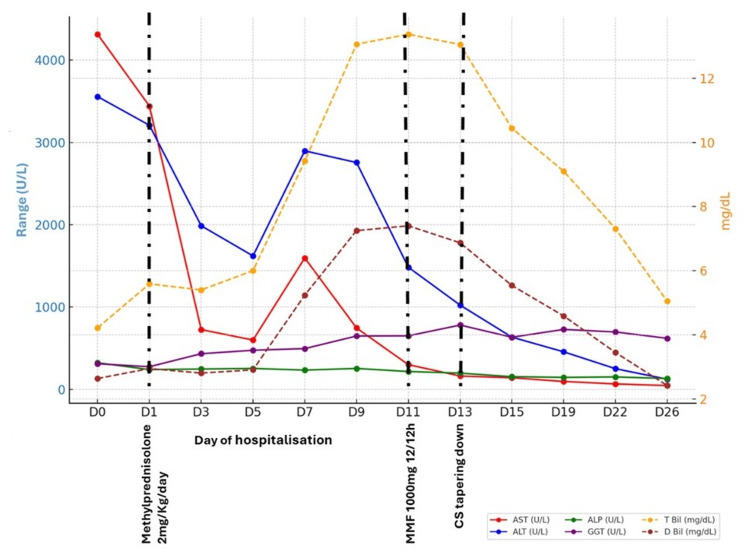
Longitudinal levels of serum ALT, AST, GGT, ALP, and bilirubin during hospitalization ALP, alkaline phosphatase; ALT, alanine aminotransferase; AST, aspartate aminotransferase; Bil, bilirubin; GGT, gamma-glutamyl transferase; CS, corticosteroid; MMF, mycophenolate mofetil

One week after admission, we observed a paradoxical biochemical response: an improvement in AST and ALT to grade 3 but a progressive worsening of hyperbilirubinemia and GGT to grade 4 (Figure 2). MMF at 1000 mg twice daily was initiated, considering CS refractoriness. One week after introducing MMF and considering liver parameters improvement, we started tapering down CS (Figure 2).

**Figure 3:**
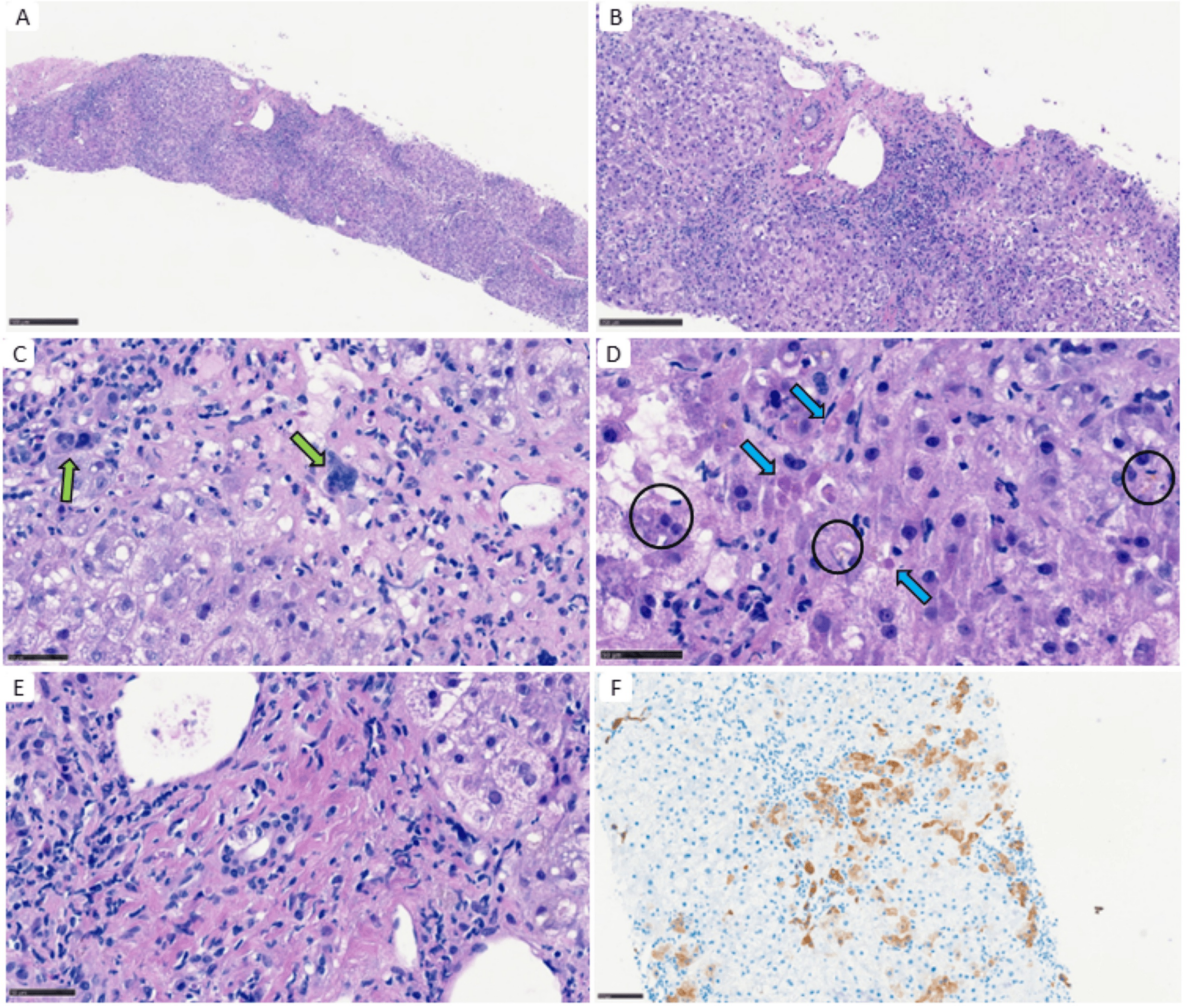
Percutaneous liver biopsy A: Trabecular collapse and moderate-to-marked inflammatory infiltrate in both periportal and centrilobular regions (HE, 4x). B: Interface hepatitis (HE, 10x). C: Hepatocyte degeneration, including multinucleated cells (green arrow; HE, 40x). D: Acidophilic bodies (blue arrow) and signs of hepatocanalicular cholestasis (circle) (HE, 40x). E: Bile ducts exhibited cytoplasmic vacuolation, nuclear overlap, and irregular nuclear contours, consistent with bile duct injury (HE, 40x). F: Cytokeratin 7 highlighted hepatocytes with intermediate phenotype (cytokeratin 7 immunostain, 20x).

A percutaneous liver biopsy was performed 13 days post-admission. Analysis revealed a moderate to marked mixed inflammatory infiltrate in periportal and centrilobular regions and confluent necrosis. Masson’s trichrome stain demonstrated mild periportal and “chicken-wire” centrilobular fibrosis (Figure 3). Histological findings were consistent with pembrolizumab-induced hepatitis, presenting a mixed pattern of hepatocellular and cholestatic injury.

Clinically, both jaundice and peripheral edema resolved and the patient remained asymptomatic. Twenty-six days after admission, AST, ALT, and ALP levels decreased to grade 1 (AST: 47 U/L, ALT: 123 U/L, and ALP: 133 U/L). GGT and hyperbilirubinemia remained elevated at grade 3 (GGT: 621 U/L, total bilirubin: 5.05 mg/dL, and direct bilirubin: 2.42 mg/dL). Albumin and INR levels were within normal limits. Given the substantial laboratory improvement and absence of liver failure signs or symptoms, the patient was discharged on MMF 1000 mg twice daily, prednisolone 60 mg once daily, and cotrimoxazole 960 mg three times a week. All potentially hepatotoxic medications were discontinued.

Due to the clinical complexity, diagnosis and treatment were discussed in our institutional Mult'iTox group, which includes specialists from different areas to support the multidisciplinary discussion and management of the most serious irAEs. 

**Figure 4:**
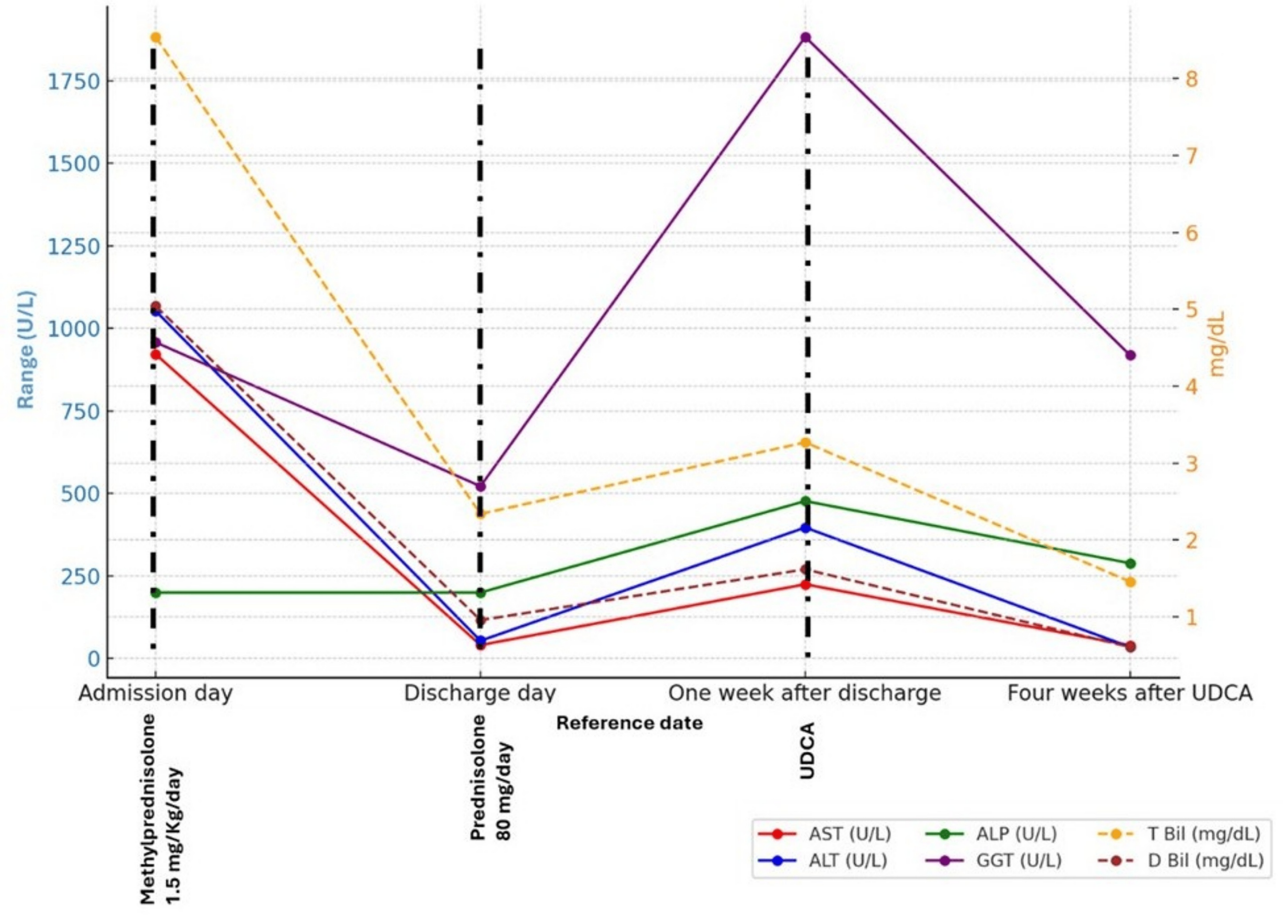
Longitudinal levels of serum ALT, AST, GGT, ALP, and bilirubin during the second hospitalization and respective evolution after discharge ALP, alkaline phosphatase; ALT, alanine aminotransferase; AST, aspartate aminotransferase; Bil, bilirubin; GGT, gamma-glutamyl transferase; UDCA, ursodeoxycholic acid

One week after discharge, the patient was readmitted due to a hepatitis relapse, evidenced by grade 4 elevations in AST and ALT (922 U/L and 1053 U/L, respectively), grade 3 hyperbilirubinemia (total and direct bilirubin levels of 8.54 and 5.04 mg/dL), and GGT (958 U/L) (Figure 4). Despite the patient’s denial of medication noncompliance, he confirmed alcohol reconsumption, which would explain these values. INR remained within normal limits, and hypoalbuminemia was mild (3.8 g/dL). No clinical signs or symptoms of liver failure were observed. The patient was managed with intravenous methylprednisolone at 1.5 mg/kg/day, resulting in improvement of liver function tests to grade 1 level (AST: 41 U/L, ALT: 54 U/L, total and direct bilirubin: 2.34 and 0.96 mg/dL, ALP: 200 U/L), except for GGT, which remained elevated at grade 3 (522 U/L) (Figure 4). The patient was discharged after an 18-day hospitalization on a tapering CS regimen (prednisolone 80 mg daily on the day of discharge). It should be noted that hospitalization was prolonged due to an intercurrent nosocomial left leg cellulitis, which resolved following empiric antibiotic therapy.

Following this second discharge, the patient experienced a re-aggravation of AST and ALT to grade 3 (225 U/L and 397 U/L, respectively), grade 3 hyperbilirubinemia (total and direct bilirubin of 3.27 and 1.62 mg/dL) and grade 4 GGT elevation (1883 U/L) (Figure 4). As a result, UDCA 750 mg (13 mg/kg/day) once daily was initiated. After four weeks of treatment, AST, and ALT normalized (AST: 40 U/L and ALT: 35 U/L), and hyperbilirubinemia decreased to grade 1 (total and direct bilirubin: 1.46 mg/dL and 0.61 mg/dL, respectively). Still, GGT only improved for a grade 3 elevation (919 U/L) (Figure 4). At that time, the patient maintained triple therapy with the aforementioned dose of UDCA, prednisolone 60 mg/day (1 mg/Kg/day), and MMF 1000 mg twice daily.

**Figure 5:**
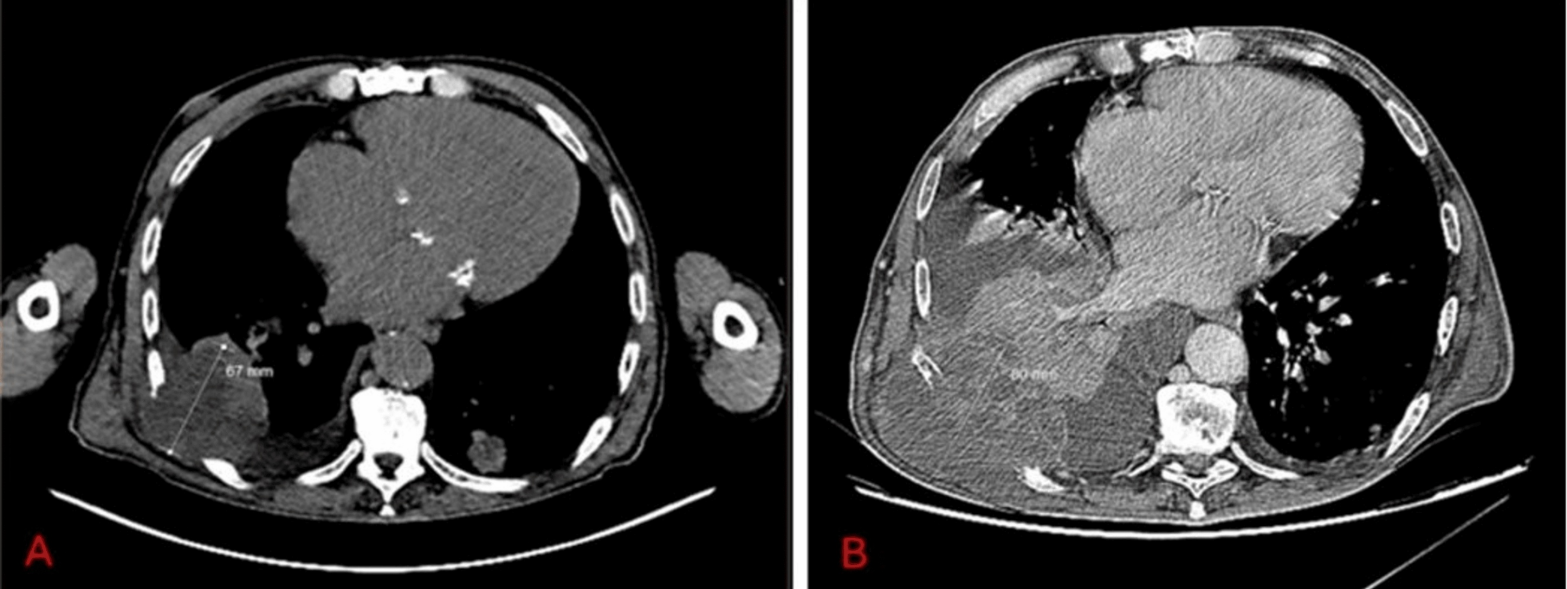
Comparative images from computed tomography scans performed at treatment baseline (A) and three months after treatment discontinuation (B), evidencing disease progression according to the RECIST criteria A: target lesion measuring 67 mm; B: target lesion measuring 80 mm + new lesions on lymph nodes and a new onset pleural effusion.

A magnetic resonance cholangiopancreatography excluded relevant hepatobiliary lesions. Regarding treatment outcomes, the disease has progressed three months after treatment discontinuation (Figure 5). However, due to the severity of ChILI and CS maintenance dose, it was neither possible to rechallenge immunotherapy nor to start chemotherapy. Therefore, the patient is under surveillance.

## Discussion

During treatment with ICI, any elevation in liver enzymes should prompt evaluation for possible confounding factors, including viral reactivation, autoimmune reactions, or alcohol abuse [1,7]. However, the potential for an irAE should be continuously suspected to avoid delays in diagnosis and initiation of appropriate treatment. Initial assessment should involve non-invasive testing, with liver biopsy reserved for cases of standard treatment refractoriness or when the diagnosis remains inconclusive, as a correlation between biological markers and histopathology has been documented [1,7]. This was our conduct during the presented case.

From a pathophysiological perspective, pembrolizumab is associated with both hepatocellular and cholestatic hepatitis, as anti-PD-L1 therapy promotes direct CD8+ T-cell cytotoxicity against hepatocytes and cholangiocytes [1,8]. Histologically, there are no pathognomonic features of ICI-induced hepatitis [7]. Nonetheless, panlobular hepatitis with a prominent peri-venular infiltrate and endotheliitis with spotty necrosis has been associated with the clinical hepatocellular pattern [3,4]. In contrast, injury predominantly affecting bile ducts is more frequently observed, though not exclusive, to the cholestatic pattern. Immunostaining showing low CD20+/CD3+ and CD4+/CD8+ cell ratios among infiltrating lymphocytes can help differentiate irAEs from autoimmune hepatitis or idiosyncratic drug-induced liver injury [3,4].

In our case, a mixed histological pattern was found. It should be noted that alcoholic habits may have contributed to the cholestatic pattern found. Additionally, in a patient with a prior diagnosis of chronic liver disease, albeit mild and well-controlled, the initiation of pembrolizumab was sufficient to account for the severity of the diagnosed hepatitis, as well as its refractoriness to the standard treatment. Since alcohol use is considered a risk factor for ChILI, as in other forms of drug-induced liver injury, opting for more frequent monitoring of liver tests might prevent the development of higher grades of hepatotoxicity if treatment is suspended earlier [9]. A lower threshold for suspending immunotherapy might be considered in those cases. 

Current dosing recommendations of CS in ChILI are inferred from protocols for other irAEs and autoimmune hepatitis management [7]. The great heterogeneity in the clinical response to different treatment options, including vigilance, oral versus intravenous CS, and alternative therapies, supports the need for a multidisciplinary approach to treatment decisions [7]. 

Although there is evidence that CS is an appropriate treatment for immunotherapy-induced hepatitis, in cases where optimal response is not achieved, there should be a low threshold for considering other therapeutic options. For refractory hepatitis, as seen in our case, European guidelines recommend MMF as a first-line therapy for CS-resistant ChILI, although evidence from prospective studies is lacking [2]. While AZA is often preferred for autoimmune hepatitis, it is not clear if it is more productive than MMF for CS-refractory ChILI [7]. Due to the limited number of reported cases of CS-resistant hepatitis requiring second-line immunosuppressors, consensus on the optimal choice of a second immunomodulator remains elusive [1,7]. Additionally, clinicians should remain vigilant for opportunistic infections, promptly initiating appropriate treatment [7].

In cases of cholestatic ChILI that resemble primary sclerosing cholangitis, which is typically managed with UDCA rather than CS, introducing UDCA as a treatment option for ChILI may be considered. Beyond its cytoprotective effects in cholestasis, UDCA might also serve as an immunomodulator by countering CD8+ T-lymphocyte-associated autoimmunity in pembrolizumab-induced cholestasis [1,4]. In our case, UDCA was considered following fluctuations in cholestasis parameters despite dual immunosuppressant therapy. The mixed histological pattern and the microscopic biliary duct lesions further supported UDCA use. The sustained clinical response after four weeks of treatment demonstrated its efficacy. 

The timing of when the liver biopsy was performed, after starting CS, is one of the potential limitations of this clinical report. Nevertheless, inflammatory cells around the portal tract and microscopic lesions in the biliary ducts were evident, enabling the diagnosis. While the follow-up period was short, the primary aim was to illustrate the complexity of managing pembrolizumab-induced and CS-refractory hepatitis, rather than to assess the impact of this event on long-term disease course.

## Conclusions

Immunotherapy can cause severe liver injury. This can be compounded in the setting of ongoing alcohol consumption and already existing chronic liver injury from alcohol use. The choice of treatment may depend on the severity of hepatitis, the patient's performance status, comorbidities, and availability of potential drugs along with their anticipated adverse events. CS therapy is the first-line treatment.

Physicians should be vigilant for the possibility of relapse during weaning of CS, as well as for refractoriness to them. A multidisciplinary approach is crucial in managing CS-refractory ChILI. This approach should involve ongoing discussions regarding treatment selection, monitoring clinical evolution, and consideration of alternative immunosuppressants. Careful assessment of potential complications, including disease progression from treatment discontinuation, and the absence of safety criteria for reintroducing antitumor medication should be considered. 
